# Evaluating overweight and obesity prevalence in survivors of childhood brain tumors: a systematic review protocol

**DOI:** 10.1186/s13643-017-0439-1

**Published:** 2017-03-03

**Authors:** Kuan-Wen Wang, Adam Fleming, Sheila K. Singh, Laura Banfield, Russell J. de Souza, Lehana Thabane, M. Constantine Samaan

**Affiliations:** 1 Department of Pediatrics, McMaster University, 1280 Main Street West, HSC-3A57, Hamilton, Ontario L8S 4K1 Canada; 2 Division of Pediatric Endocrinology, McMaster Children’s Hospital, Hamilton, Ontario Canada; 3 Medical Sciences Graduate Program, McMaster University, Hamilton, Ontario Canada; 4 Division of Pediatric Hematology/Oncology, McMaster Children’s Hospital, Hamilton, Ontario Canada; 5 Division of Neurosurgery, Department of Surgery, McMaster Children’s Hospital, Hamilton, Ontario Canada; 6 McMaster Stem Cell and Cancer Research Institute, McMaster University, Hamilton, Ontario Canada; 7 Health Sciences Library, McMaster University, Hamilton, Ontario Canada; 8 Department of Health Research Methods, Evidence and Impact, McMaster University, Hamilton, Ontario Canada; 9 Department of Anesthesia, McMaster University, Hamilton, Ontario Canada; 10 Centre for Evaluation of Medicines, St. Joseph’s Health Care, Hamilton, Ontario Canada; 11 Biostatistics Unit, St Joseph’s Healthcare, Hamilton, Ontario Canada

**Keywords:** Systematic review protocol, Protocol, Obesity, Childhood brain tumor, Cancer survivorship

## Abstract

**Background:**

Overweight and obesity are well-known risk factors for cardiometabolic diseases including hypertension, myocardial infarction, stroke, and type 2 diabetes in the general population. Survivors of childhood brain tumors (SCBT) are at risk of premature mortality, and recent evidence suggests that these cardiometabolic diseases are potential emerging determinants of survival and quality of life. Therefore, the rates of overweight and obesity in this population need to be examined to assess their impact on outcomes. The objective of this systematic review is to examine the prevalence of overweight and obesity in SCBT. The secondary aim of this review is to evaluate whether SCBT have higher adiposity compared to the general population.

**Methods:**

Searches will be conducted in MEDLINE, CINAHL, EMBASE, Cochrane Database of Systematic Reviews, Cochrane Central Register of Controlled Trials, PubMed, and Database of Abstracts of Reviews of Effect. For gray literature, we will search ProQuest Dissertations and Theses A&I and Web of Science. Two reviewers will independently screen all articles against predetermined eligibility criteria and complete data abstraction, risk of bias, and quality assessments. The primary outcome includes the prevalence of overweight or obesity. The secondary outcomes involve waist-to-hip ratio, waist-to-height ratio, body fat percentage, and skinfold thickness. Meta-analysis will be performed when two or more studies with similar design, populations, and outcomes are available.

**Discussion:**

This review will summarize current data on the prevalence of overweight and obesity in SCBT. This will help the development of an understanding of the scale of overweight and obesity in this population and guide the design of interventions that will improve outcomes.

**Systematic review registration:**

PROSPERO CRD42016051035

**Electronic supplementary material:**

The online version of this article (doi:10.1186/s13643-017-0439-1) contains supplementary material, which is available to authorized users.

## Background

Recent advances in the management of pediatric brain tumors have significantly improved survival rates [1, 2]. However, the new record longevity noted in Survivors of Childhood Brain Tumors (SCBT) is being hindered by the emergence of new comorbidities including cardiometabolic diseases like hypertension, myocardial infarction, stroke, and type 2 diabetes [3–13]. The current global overweight and obesity epidemic has been blamed for the rise of these cardiometabolic disorders in the general population, but the scale of overweight and obesity and its role in driving adverse outcomes in survivors is unknown.

Of note, SCBT have several risk factors that predispose them to overweight and obesity. These include impaired satiety signals, lower physical activity, impaired mobility and coordination, pain, disrupted sleep, mental health concerns, pituitary hormonal deficiencies, and medications [14–17]. To further understand the contribution of overweight and obesity to cardiometabolic risk in SCBT, there is a need to determine its scale in SCBT. This will inform the design of interventions to target overweight and obesity and their risk factors to improve cardiometabolic outcomes, quality of life, and survival rates in this population.

In this systematic review, the epidemiological data on the prevalence of overweight and obesity in SCBT will be evaluated. The primary aim of this review is to determine whether SCBT have higher rates of overweight or obesity compared to non-cancer counterparts. The secondary aim of this review is to evaluate whether SCBT have higher adiposity compared to the general population.

## Methods

This protocol is developed according to the Preferred Reporting Items for Systematic Review and Meta-Analysis-Protocols (PRISMA-P) statement [18, 19] (Additional file 1).

### Literature search

Searches will be conducted in MEDLINE, CINAHL, EMBASE, Cochrane Database of Systematic Reviews, Cochrane Central Register of Controlled Trials, PubMed, and Database of Abstracts of Reviews of Effect. The following concepts along with their synonyms will be used in the search: pediatric, brain tumors, overweight/obesity, and survivors. A search strategy will be developed in consultation with a senior health sciences librarian with expertise in systematic reviews. We will not set any restrictions on publication date, but will restrict our search to English language publications. A full search strategy for MEDLINE is reported in Table 1.

**Table 1:** Search strategy for MEDLINE

#	Searches
1	exp Child/
2	child*.ab,ti,kf.
3	p?ediatric*.ab,ti,kf.
4	exp Adolescent/
5	adolescen*.ab,ti,kf.
6	youth*.ab,ti,kf.
7	teen*.ab,ti,kf.
8	kid*.ab,ti,kf.
9	1 or 2 or 3 or 4 or 5 or 6 or 7 or 8
10	exp Brain Neoplasms/
11	exp Neuroectodermal Tumors/
12	exp Glioma/
13	glioma*.ab,ti,kf.
14	astrocytoma*.ab,ti,kf.
15	oligoastrocytoma*.ab,ti,kf.
16	astroglioma*.ab,ti,kf.
17	glioblastoma*.ab,ti,kf.
18	craniopharyngioma*.ab,ti,kf.
19	ependymoma*.ab,ti,kf.
20	subependymoma*.ab,ti,kf.
21	ependymoblastoma*.ab,ti,kf.
22	ganglioglioma*.ab,ti,kf.
23	medulloblastoma*.ab,ti,kf.
24	exp Germinoma/
25	germinoma*.ab,ti,kf.
26	Meningioma/
27	meningioma*.ab,ti,kf.
28	oligodendroglioma*.ab,ti,kf.
29	exp Neurofibromatoses/
30	neurofibromatos*.ab,ti,kf.
31	PNET*.ab,ti,kf.
32	neurocytoma*.ab,ti,kf.
33	choroid plexus papilloma*.ab,ti,kf.
34	((brain or central nervous system or CNS or brainstem or brain stem or cerebel* or cerebr* or hypothalam* or ventric* or intracranial or midline or choroid plexus or infratentorial or supratentorial or neuroectoderm* or germ cell*) adj5 (tumo?r* or neoplasm* or cancer*)).ab,ti,kf.
35	10 or 11 or 12 or 13 or 14 or 15 or 16 or 17 or 18 or 19 or 20 or 21 or 22 or 23 or 24 or 25 or 26 or 27 or 28 or 29 or 30 or 31 or 32 or 33 or 34
36	exp Obesity/
37	obes*.ab,ti,kf.
38	Overweight/
39	over weight.ab,ti,kf.
40	overweight.ab,ti,kf.
41	Body Weight/
42	exp Body Composition/
43	(body adj3 (mass* or size* or composition*)).ab,ti,kf.
44	(fat* adj3 (mass* or body or abdominal* or intra-abdominal* or viscera* or subcutane* or hepatic* or liver* or intramuscular* or intramyocellular*)).ab,ti,kf.
45	BMI*.ab,ti,kf.
46	Weight Gain/
47	exp “Body Weights and Measures”/
48	Anthropometry/
49	anthropometr*.ab,ti,kf.
50	grow*.ab,ti,kf.
51	overnutrition*.ab,ti,kf.
52	over nutrition*.ab,ti,kf.
53	malnutrition*.ab,ti,kf.
54	waist-height ratio*.ab,ti,kf.
55	waist to height ratio*.ab,ti,kf.
56	adipos*.ab,ti,kf.
57	((waist* or hip* or abdominal*) adj3 circumference*).ab,ti,kf.
58	(weight* adj3 (gain* or change* or fluctuat*)).ab,ti,kf.
59	waist-hip ratio*.ab,ti,kf.
60	waist to hip ratio*.ab,ti,kf.
61	skinfold thickness*.ab,ti,kf.
62	36 or 37 or 38 or 39 or 40 or 41 or 42 or 43 or 44 or 45 or 46 or 47 or 48 or 49 or 50 or 51 or 52 or 53 or 54 or 55 or 56 or 57 or 58 or 59 or 60 or 61
63	Survivors/
64	“Adult Survivors of Child Adverse Events”/
65	Disease-Free Survival/
66	surviv*.ab,ti,kf.
67	remission*.ab,ti,kf.
68	((post or off or after) adj5 (treatment* or therap*)).ab,ti,kf.
69	((treatment* or therap* or cancer* or disease* or event* or progression*) adj5 free).ab,ti,kf.
70	63 or 64 or 65 or 66 or 67 or 68 or 69
71	9 and 35 and 62 and 70
72	limit 71 to english language

To identify grey literature, we will search ProQuest Dissertations and Theses A&I and Web of Science. The search in the latter database will be limited to “Conference Proceedings Citation Index-Science-1990-present.” We will then search for relevant publications from the first and last authors of the relevant conference abstracts to identify articles originating from the work presented in the abstracts. The reference lists of eligible studies and relevant reviews will also be searched to identify any additional studies. Searches will be updated to capture recent publications by setting publication date restrictions.

The search results will be de-duplicated in EndNote X7 [20] and then exported into an excel file to screen for eligible titles and abstracts. The full texts of relevant records will then be retrieved to screen against the eligibility criteria.

### Study selection and eligibility criteria

Two independent reviewers, who will meet after each stage to resolve conflicts and achieve consensus, will screen the title and abstract of each record. A third reviewer will be consulted when disagreements persist. The two reviewers will then independently screen the full text of the relevant studies identified from the title and abstract screening.

**Fig. 1:**
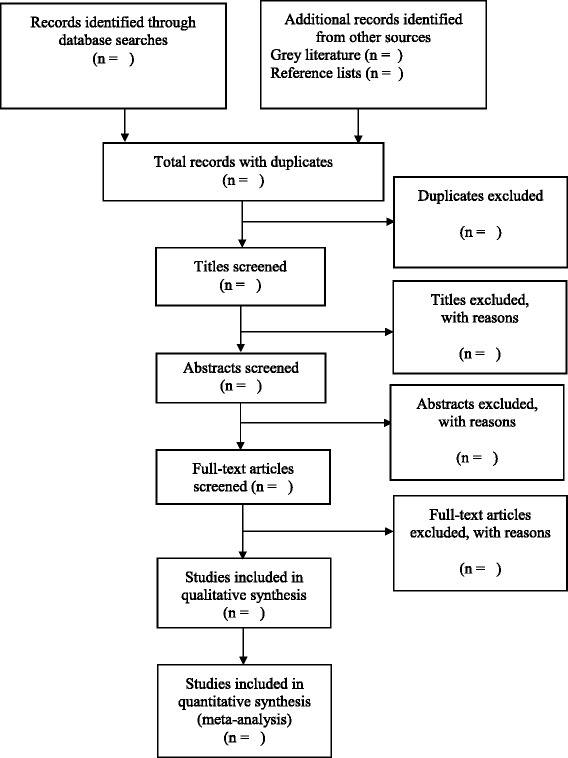
PRISMA flow diagram

This review will include SCBT diagnosed under 18 years of age. The following eligibility criteria will be applied: (1) Primary research articles with observational study design including longitudinal cohort, cross-sectional, or case-control studies. (2) Sample size of ≥10 patients as previously described [21]. (3) Assessment of prevalence of overweight or obesity and/or body composition using measures including Body Mass Index (BMI), BMI z-score, BMI percentile, waist-to-hip ratio, waist-to-height ratio, body fat, and skinfold thickness. The screening process and results will be reported in a PRISMA flow diagram, as previously described [22–24] (Fig. 1).

### Data collection

We developed a data abstraction form that will be piloted by two reviewers on two eligible studies. Comments will then be incorporated to finalize the form for this specific systematic review. The abstracted data will include publication information of title, authors’ names, journal name, year of publication, as well as the city and country of publication. We will also collect study details including setting, study design, eligibility criteria, sample size, study duration, and funding source. Outcome measures, primary findings, and conclusions will be collected as well.

We will extract survivors’ characteristics including age at diagnosis of brain tumor, age at study enrollment, and sex. We will also extract brain tumor details including brain tumor type and location and treatment details such as treatment period, duration since treatment completion, and types of treatments received including radiotherapy, chemotherapy, and surgery or combination therapies with these modalities. If the study has a non-cancer comparison group, we will document the type and source of non-cancer controls used and abstract the same data except for tumor- and treatment-related variables.

Two reviewers will perform data abstraction independently, followed by a discussion to resolve discrepancies. A third reviewer will intervene to resolve persisting differences. In studies that report the data from multiple cancer types as aggregates, data specific to the brain tumor group will be extracted either through published subgroup data or by contacting the research team to acquire the data. We will also contact the corresponding authors of a published work in attempts to obtain any missing data.

The primary outcome for this review is the prevalence of overweight or obesity estimated by BMI, BMI z-score, or BMI percentile. Secondary outcomes include waist-to-hip ratio, waist-to-height ratio, body fat percentage, and skinfold thickness.

### Risk of bias and quality assessment

Two reviewers will independently assess the risk of bias of the eligible studies using the Newcastle-Ottawa Scale (NOS) for observational studies [25]. The NOS will be adapted from its original version by considering a previously used modified version [26], so that the scale is specific to this review. The reviewers will meet and discuss their decisions to include articles and to resolve any disagreement. In the case of persisting conflict, a third reviewer will be consulted.

This adapted NOS evaluates five items pertaining to risk of bias due to sample selection and classification (two items), confounding factors (one item), missing data (one item), and measurement errors (one item). For each item, the risk of bias is rated on a scale of 0 (high risk of bias), 1–2 (moderate risk of bias), and 3 (low risk of bias). The risk of bias is rated as unclear if not enough information is provided. Descriptions with examples for each level of risk of bias are provided (Additional file 2).

The overall risk of bias is rated as low when all five items have low risk of bias or high when one or more items have high risk of bias. The overall risk of bias is considered to be moderate when not all items have low risk of bias, but there are no items with high risk of bias. If one of the items is rated as unclear, the overall risk of bias will be reported as unclear as well.

Furthermore, we will use the Grading of Recommendations, Assessment, Development, and Evaluation (GRADE) guideline [27] to evaluate the overall quality of evidence including the risk of bias, inconsistency, indirectness, imprecision, and publication bias to determine the overall quality of evidence for each outcome.

### Statistical analysis

We will perform meta-analysis if two or more studies of similar design and population characteristics can be identified for each outcome. We expect high heterogeneity across studies. The possible sources of heterogeneity include age at diagnosis, duration and types of treatment, and brain tumor type and location. Therefore, we will perform meta-analysis using a random effects model if more than ten studies are eligible and will perform both random effects and fixed effects models if less than ten studies are identified [28].

Dichotomous and continuous outcomes will be reported as pooled odds ratio and standardized mean difference with 95% confidence intervals, respectively. In studies where multiple measurements are done, we will include the outcomes measured with the longest follow-up reported.

Both inconsistency index (*I* ^*2*^) and *P* values from the chi-square test for homogeneity will be considered to determine the level of heterogeneity among the included studies. The threshold set by the Cochrane Collaboration will be used to interpret *I* ^*2*^, with >75% representing considerable heterogeneity. A *P* value of <0.10 will be used to determine statistical significance [29]. If meta-analysis is not appropriate, heterogeneity will be evaluated by describing and comparing the study samples, methods, and designs across studies. We will perform subgroup meta-analysis by sex and receipt of radiotherapy, chemotherapy, and surgery or combination therapies with these modalities if appropriate, as it has been reported that female SCBT are at higher risk of obesity than males [7, 8, 11]. In addition, to test the impact of outliers and studies with high risk of bias on the results, we will perform sensitivity analysis by excluding these studies if ten or more studies can be identified for an outcome.

To maintain the power of the results, we will not perform sensitivity analyses if less than ten studies are eligible. If ten or more studies are identified, we will use a contour-enhanced funnel plot to investigate publication bias [30]. The plot asymmetry will be determined by Egger’s test and visual inspection [30]. Otherwise, we will estimate publication bias based on the number of relevant conference abstracts that did not have published articles originating from the work presented in the abstracts [31].

We will use Review Manager Version 5.3 Software (RevMan 5.3) [32] to conduct the meta-analysis. If Egger’s test is appropriate, Comprehensive Meta-Analysis Software Version 3 (CMA 3.0) will be used instead [33]. A comprehensive table for summary of findings with narrative description will be reported when a meta-analysis is not appropriate.

We will report the results of this systematic review in accordance with the Preferred Reporting Items for Systematic Reviews and Meta-Analyses (PRISMA) guidelines using the PRISMA checklist [22, 23]. We will also document the date and reasons for any amendments to the protocol.

## Discussion

While record numbers of children are surviving the diagnosis of brain tumors, this survival is burdened by the high rate of comorbidities and premature mortality [10, 12, 34]. To improve the quality of the cure, detailed understanding of the factors driving comorbidities in SCBT is likely to provide therapeutic entry points to improve outcomes.

Recent evidence suggests that new emerging risk factors may be contributing to mortality in this population. With increasing longevity, SCBT are at risk of type 2 diabetes and cardiovascular diseases that appear relatively early in life [3–6, 9]. This argues for a premature aging process, whereby diseases of old age are appearing earlier in life in SCBT. This may indicate that similar overweight or obesity levels may have a disproportionately negative impact on SCBT when compared to the general population, and interventions are needed to stem the occurrence of overweight and obesity and reduce their burden in survivors. Notable limitations of this systematic review include the restriction of the search strategy to English language publications only, as this may lead to missing information from non-English literature. In addition, if the heterogeneity of the studies is high, this will preclude the performance of a meta-analysis. Nevertheless, this review will identify gaps in knowledge and inform better clinical practice in identifying overweight and obesity and will help inform the need for specifically designed interventions to tackle overweight and obesity in SCBT and improve outcomes.

## Additional files

Additional file 1: PRISMA-P checklist. This checklist includes recommended items to address in a systematic reviews protocol and where they are reported in this protocol. (DOCX 36 kb)
Additional file 2: Adapted version of a modified Newcastle-Ottawa Scale (NOS) to evaluate overweight and obesity in survivors of childhood brain tumors. This form demonstrates the adapted version of the NOS to evaluate risk of bias of the included observational studies in this systematic review. (DOCX 17 kb)

## Abbreviations

BMI: Body Mass Index
CINAHL: Cumulative Index to Nursing and Allied Health Literature
CMA 3.0: Comprehensive Meta-Analysis Software Version 3.0
EMBASE: Excerpta Medica dataBASE
GRADE: Grading of Recommendations, Assessment, Development and Evaluation
MEDLINE: Medical Literature Analysis and Retrieval System Online
NOS: Newcastle-Ottawa Scale
PRISMA: Preferred Reporting Items for Systematic Reviews and Meta-Analyses
PRISMA-P: Preferred Reporting Items for Systematic Review and Meta-Analysis-Protocols
RevMan 5.3: Review Manager Version 5.3 Software
SCBT: Survivors of Childhood Brain Tumors
