# Supplementary material for: Evaluating overweight and obesity prevalence in survivors of childhood brain tumors: a systematic review protocol
Source: Syst Rev. 2017 Mar 3;6:43. doi: 10.1186/s13643-017-0439-1 (PMC5335753; doi:10.1186/s13643-017-0439-1)
Supplement: Additional file 2: — Adapted version of a modified Newcastle-Ottawa Scale (NOS) to evaluate overweight and obesity in survivors of childhood brain tumors. This form demonstrates the adapted version of the NOS to evaluate risk of bias of the included observational studies in this systematic review. (DOCX 17 kb) [file 13643_2017_439_MOESM2_ESM.docx]

**Additional file 2:** Adapted version of a modified Newcastle-Ottawa Scale to evaluate overweight and obesity prevalence in survivors of childhood brain tumors

| 0 = Definitely no |
| --- |
| 1 = Mostly no |
| 2 = Mostly yes |
| 3 = Definitely yes |
| Unclear = not enough information provided |

Domain 1: Selection

**Is the selection of study sample consecutive and representative of the population of interest?**

Low risk of bias: random sampling and consecutive recruitment from a representative population.

Moderate risk of bias: random sampling and consecutive recruitment from a non-representative population

High risk of bias: non-random sampling, non-consecutive recruitment

Recruitment is consecutive when explicit statement is provided or when all (or a random sample of) subjects during a given date range are included.

**If there is a comparison group, are the selection of non-cancer control and classification of brain tumor status appropriate?**

Low risk of bias: the non-cancer control is selected from the same community as the childhood brain tumors survivors and the brain tumor status is determined by medical records

Moderate risk of bias: the non-cancer control is selected from a different source and/or the brain tumor status is self-reported

High risk of bias: there is no description for the selection of non-cancer control and/or how classification is done

Domain 2: Comparability

**Does the study identify and adjust for confounding factors in the analysis?**

Low risk of bias: possible confounding factors are identified and adjusted for

Moderate risk of bias: possible confounding factors are identified but not adjusted for

High risk of bias: no confounding factors are identified when they are clearly present

Possible confounding factors include age, sex, the location and histology of brain tumors, types of treatment received for the brain tumors, years of survival, and the presence of other comorbidities such as endocrinolpathies.

Domain 3: Missing data

**Are incomplete/missing data addressed adequately?**

Low risk of bias: there are ≤10% incomplete/missing data

Moderate risk of bias: there are ≤25% incomplete/missing data and appropriate methods of addressing them are specified

High risk of bias: there are >25% incomplete/missing data or ≤25% incomplete/missing data and no methods are used to address them

Domain 4: Outcome

**Are outcome measuring methods appropriate?**

Low risk of bias: brain tumor treatment modalities are obtained from medical records and anthropometric measurements are done in duplicate with appropriate/justified methods

Moderate risk of bias: brain tumor treatment modalities are self-reported and/or anthropometric measurements are not done in duplicate but methods are appropriate or justified

High risk of bias: the methods used for anthropometric measurements are inappropriate or unjustified
